# Supplementary figures and images for: Algorithm for semi-automatic detection of insulin granule exocytosis in human pancreatic β-cells
Source: Heliyon. 2024 Sep 27;10(19):e38307. doi: 10.1016/j.heliyon.2024.e38307 (PMC11483283; doi:10.1016/j.heliyon.2024.e38307)

**A**

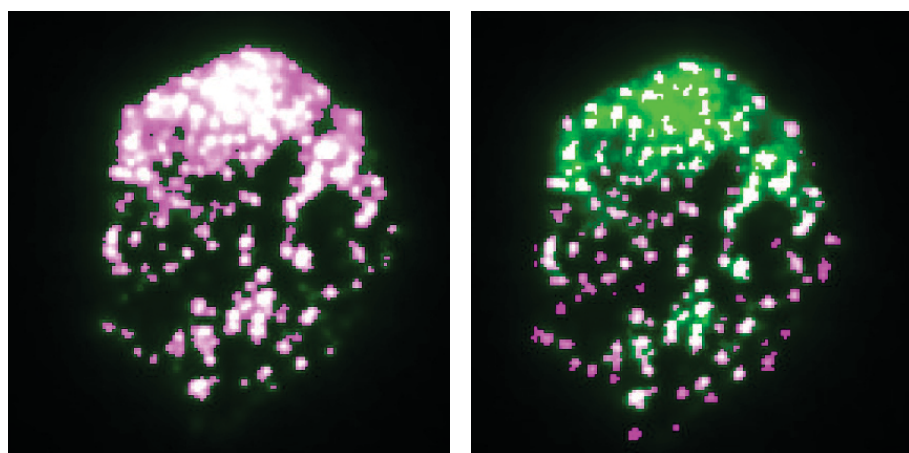

**B**

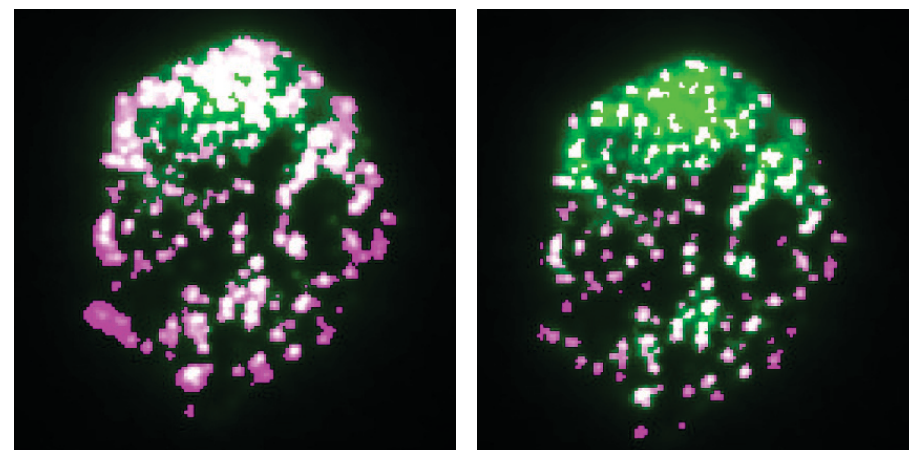

**Supplementary figure 1**

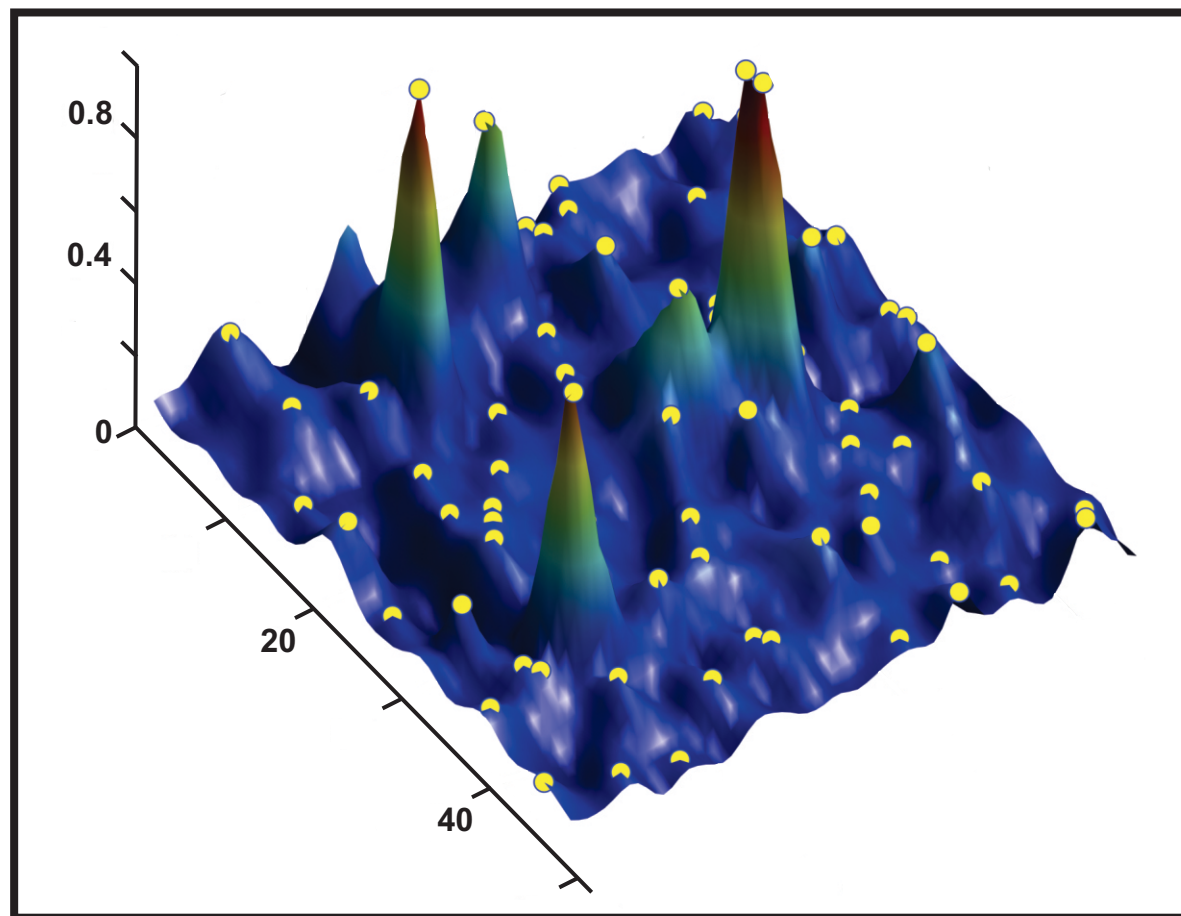

**Supplementary figure 2**

Supplement: Multimedia component 1 [file mmc1.pdf]
